# Multispacer Sequence Typing Relapsing Fever Borreliae in Africa

**DOI:** 10.1371/journal.pntd.0001652

**Published:** 2012-06-05

**Authors:** Elbir Haitham, Gregory Gimenez, Cheikh Sokhna, Kassahun Desalegn Bilcha, Jemal Ali, Stephen C. Barker, Sally J. Cutler, Didier Raoult, Michel Drancourt

**Affiliations:** 1 Unité de Recherche sur les Maladies Infectieuses et Tropicales Emergentes (URMITE), UMR CNRS 6236 IRD 198, Faculté de Médecine, Méditerranée Infection, Aix-Marseille Université, Marseille, France; 2 Unité de Recherche sur les Maladies Infectieuses et Tropicales Emergentes (URMITE), UMR CNRS 6236 IRD 198, Méditerranée Infection, Aix-Marseille Université, Dakar, Senegal; 3 College of Medicine and Health Sciences, University of Gondar, Gondar, Ethiopia; 4 Parasitology Section, School of Chemistry and Molecular Bioscience, University of Queensland, Brisbane, Australia; 5 School of Health, Sports and Bioscience, University of East London, London, United Kingdom; University of California San Diego School of Medicine, United States of America

## Abstract

**Background:**

In Africa, relapsing fevers are neglected arthropod-borne infections caused by closely related *Borrelia* species. They cause mild to deadly undifferentiated fever particularly severe in pregnant women. Lack of a tool to genotype these *Borrelia* organisms limits knowledge regarding their reservoirs and their epidemiology.

**Methodology/Principal Findings:**

Genome sequence analysis of *Borrelia crocidurae, Borrelia duttonii* and *Borrelia recurrentis* yielded 5 intergenic spacers scattered between 10 chromosomal genes that were incorporated into a multispacer sequence typing (MST) approach. Sequencing these spacers directly from human blood specimens previously found to be infected by *B. recurrentis* (30 specimens), *B. duttonii* (17 specimens) and *B. crocidurae* (13 specimens) resolved these 60 strains and the 3 type strains into 13 species-specific spacer types in the presence of negative controls. *B. crocidurae* comprised of 8 spacer types, *B. duttonii* of 3 spacer types and *B. recurrentis* of 2 spacer types.

**Conclusions/Significance:**

Phylogenetic analyses of MST data suggested that *B. duttonii, B. crocidurae* and *B. recurrentis* are variants of a unique ancestral *Borrelia* species. MST proved to be a suitable approach for identifying and genotyping relapsing fever borreliae in Africa. It could be applied to both vectors and clinical specimens.

## Author Summary

In Africa, relapsing fevers are caused by four cultured species: *Borrelia crocidurae, Borrelia duttonii, Borrelia hispanica* and *Borrelia recurrentis*. These borreliae are transmitted by the bite of *Ornithodoros* soft ticks except for *B. recurrentis* which is transmitted by louse *Pediculus humanus*. They cause potentially undifferentiated fever infection and co-infection with malaria could also occur. The exact prevalence of each *Borrelia* is unknown and overlaps between *B. duttonii* and *B. crocidurae* have been reported. The lack of tools for genotyping these borreliae limits knowledge concerning their epidemiology. We developed multispacer sequence typing (MST) and applied it to blood specimens infected by *B. recurrentis* (30 specimens), *B. duttonii* (18 specimens) and *B. crocidurae* (13 specimens), delineating these 60 strains and the 3 type strains into 13 species-specific spacer types. *B. crocidurae* strains were classified into 8 spacer types, *B. duttonii* into 3 spacer types and *B. recurrentis* into 2 spacer types. These findings provide the proof-of-concept that that MST is a reliable tool for identification and genotyping relapsing fever borreliae in Africa.

## Introduction

In Africa, relapsing fevers (RF) are arthropod-borne diseases caused by four cultured species *Borrelia crocidurae, Borrelia duttonii, Borrelia hispanica* and *Borrelia recurrentis* [1]. Transmission is by the bite of *Ornithodoros* soft ticks for the first three species whereas *Pediculus humanus* louse feces transmit *B. recurrentis* [2], [3]. In Tanzania, molecular investigations of human and tick specimens further provided evidences for two additional, yet uncultured *Borrelia* species [1], [4]. Each one of the four cultured *Borrelia* species is more prevalent in one geographical area of Africa with *B. hispanica* being reported in Morocco [5], *B. crocidurae* in Senegal [6], *B. duttonii* in Tanzania [7] and *B. recurrentis* in Ethiopia [8]. However, the precise area of distribution of each *Borreli*a is unknown and may overlap as both *B. duttonii* and *B. crocidurae* have been reported in Togo and Tanzania [1], [9].

In these regions of Africa, RF was reported to be the most prevalent bacterial disease, accounting for 8.8% of febrile patients in Togo [9]. In Senegal, average incidence is 11 per 100 person-years [10]. The main clinical symptom of infection is recurrent undifferentiated fever associated with high bacteremia; RF are therefore often diagnosed as malaria and cases of malaria co-infection with have been reported [9], [11], [12]. RF are treatable by antibiotics. Severity ranges from asymptomatic to fatal, particularly if left untreated and can be associated with significant pregnancy loss or peri-natal mortality [13], [14], [15].

The African RF *Borrelia* are very closely related species as illustrated by 16S rRNA gene sequence variability ≤1% [2]. Accordingly, a previous comparison of *B. duttonii* and *B. recurrentis* genomes indicated that the two organisms formed a unique bacterial species [16]. Such a close genetic and genomic proximity challenged the development of laboratory tools for the accurate discrimination between the African RF *Borrelia* and genotyping [16]. Sequencing the 16S rRNA and the flagellin genes is unsatisfactory since African RF *Borrelia* differ by only one base in the flagllin gene sequence and have 16S rRNA gene sequence similarity above 99% [17]. Analysis of the intrergenic spacer (IGS) located between the 16S and 23S rRNA genes only explored the variability between *B. duttonii* and *B. recurrentis* [1]. Moreover, IGS sequence overlapped between one *B. duttonii* phylogenetic group and one *B. recurrentis* group [1] with a second overlap disclosed with subsequent analyses of further material [7].

We previously observed that multispacer sequence typing (MST), a PCR-sequencing-based method for bacteria genotyping, was efficient in typing otherwise homogenous bacterial species such as the plague agent *Yersinia pestis* [18] and the typhus agent *Rickettsia prowazekii* [19]. Ongoing study of the *B. crocidurae* genome in our laboratory gave us the opportunity to develop MST for African RF *Borrelia* and to deliver the proof-of-concept that MST is a suitable method for both the species identification and genotyping of RF *Borrelia* in Africa.

## Materials and Methods

### *Borrelia* strains and DNA

*B. crocidurae* Achema strain, *B. recurrentis* A1 strain and *B. duttonii* Ly strain were grown in BSK-H medium (Sigma, Saint Quentin Fallavier, France) supplemented with heat-inactivated 10% rabbit serum (Eurobio, Courtaboeuf, France). *B. recurrentis* DNA was extracted from 21 blood specimens collected in 1994 in Addis Ababa, Ethiopia Dr. S. J. Cutler (School of Health, Sports and Bioscience, University of East London, London UK). Likewise, *B. recurrentis* DNA extracted from 9 blood specimens collected in 2011 in Bahir Dah, Highlands of Ethiopia were provided by SC Barker (Parasitology section, School of Chemistry and Molecular Bioscience, University of Queensland, Brisbane, Australia) and KD Bilcha and J Ali (University of Gondar, Ethiopia). In addition, *B. duttonii* DNA extracted from **17** blood specimens collected in Mvumi, Tanzania were also provided by Dr. S. J. Cutler. *B. crocidurae* DNA was extracted from 13 blood specimens collected in 2010 in Senegal by C. Sokhna (URMITE, Dakar, Senegal) including 11 specimens from Dielmo and 2 specimens from Ndiop. DNA was extracted from these specimens using QIAamp DNA Blood mini kits (QIAGEN, Hilden, Germany) according to the manufacturer's instructions.

### Selection of intergenic spacers

The *B. crocidurae* genome (Genbank accession number CP003426–CP003465) has been sequenced and annotated in our laboratory using pyrosequencing technology on a Roche 454 GS FLX sequencer. The draft genome is comprising of one closed chromosome and scaffolds representing the plasmids. Spacer sequences extracted from *B. crocidurae* strain Achema, *B. recurrentis* strain A1 (Genbank accession number CP000993) and *B. duttonii* strain Ly (Genbank accession number CP000976) genomes using perl script software were compared using ssaha2 software [20]. Spacers were pre-selected for a 300 to 800-bp length. Pre-selected spacers were further analyzed for sequence similarity in order to exclude spacers with <0.1% interspecies sequence similarity. PCR primers were then designed using primer3 software (http://fokker.wi.mit.edu) in order to amplify the entire sequence of each of the selected spacers.

### Multispacer sequencing typing

Five microliters of *Borrelia* DNA and 10 pmol of each primer (Eurogentec, Seraing, Belgium) were added to the PCR mixture, containing 0.4 U Phusion DNA Polymerase (Finnzymes, Espoo, Finland), 4 µl of 5× Phusion HF Buffer (Finnzymes) and 0.4 µl of 10 mM dNTPs. The volume was adjusted to 24 µL by adding distilled water. Thermal cycling was performed on a 2720 DNA thermal cycler (Applied Biosystems, Courtaboeuf, France) with an initial 30-sec cycle at 98°C followed by 35 cycles consisting of 10 seconds at 98°C, 30 seconds at 58°C and 1 minute at 72°C, followed by a 10-min final extension step at 72°C. To rule out amplicon carry-over, nucleotide-free water negative control was used throughout the steps of the protocol. PCR products were purified prior to sequencing by using the Nucleo-Fast 96 PCR Kit (Macherey-Nagel, Hoerdt, France). Three microliters of the resulting DNA were added to each primer mixture comprised of 10 pmol of each primer, 4 µL water and 3 µL BigDye Terminator reaction mix (Applied Biosystems). Sequencing thermal cycling was performed on a Applied Biosystems DNA thermal cycler with an initial 5-min cycle at 96°C followed by 25 cycles consisting of 30 seconds at 96°C, 20 seconds at 55°C, and 4 minutes at 60°C, followed by a 7-min final extension step at 15°C. Sequencing products were purified using sephadex plates (Sigma-Aldrich, Saint Quentin Fallavier, France) and sequencing electrophoresis was performed on a 3130 Genetic Analyzer (Applied Biosystems).

### Sequence analysis

The nucleotide sequences were edited using ChromasPro software (www.technelysium.com.au/chromas.html). Similarities between spacers were determined after multiple alignments using the MULTALIN software [21]. MST discrimination power was calculated using the Hunter-Gaston Index [22]:
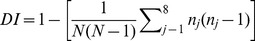
where *D* is the numerical index of discrimination, *N* is the total number of isolates in the sample population, *s* is the total number of different types, and *nj* is the number of isolates belonging to the *j*th type.

The five spacer sequences analyzed herein were concatenated and neighbor-joining phylogenetic tree was reconstructed using the maximum likelihood method in PhyML 3.0 [23]. Each particular sequence of a given spacer was assigned to a spacer type (ST) number.

### Ethics statement

This study was approved by the IFR48 Ethic Committee. All patients provided informed written consent.

## Results

### Spacer selection

Chromosome sequence alignment of the three *Borrelia* reference genomes studied herein revealed that 23 intergenic spacers that were common to all three species. Of these, five spacers fulfilled our selection criteria and were named MST2, MST3, MST5, MST6 and MST7. Use of the PCR primers listed in Table 1 to amplify each of the five spacers produced amplicons ranged from 333-bp to 738-bp and sequence reads ranging from 246-bp to 543-bp (Table 1).

**Table 1:** List of primers and genes flanking five intergenic spacers herein studied in relapsing fever *Borrelia*.

Spacers	Start End	Spacer flanking genes (5------3)	Primers	PCR product size (bp)	Spacer size (bp)
MST2	786480.. 786968	penicillin-binding protein**//**uncharacterized conserved protein	F:TTTTTGCTAAAATTAACCCTTTTCAR:CTCATTTTAATTTCCTTACCCCTA	578	487
MST3	669736.. 670279	N-acetylmuramoyl-L-alanine amidase, putative//vacuolar X-prolyl dipeptidyl aminopeptidase I	F:GCAGGTGGCTGTTAACCACTR:ATGTGGGGAATGCACTCTTT	687	543
MST5	565860.. 566397	translation elongation factor G**//**uncharacterized conserved protein	F:CCTGAGTCGATATGGGCACTR:CAACCTGACATATCTTACTCAATTCAT	653	536
MST6	494656.. 494903	tRNA-ser**//**DNA polymerase III subunits gamma and tau	F:GGGTTCGAATCCCATTTTCTR:CTCTGGGACGCCTCTTAATG	333	246
MST7	458283.. 458778	16S ribosomal RNA//hypothetical protein	F:TTCGCCACTGAATGTATTGCR:TGCCAATGTTCTTGTTGGTC	738	494

Start and end of spacer are according *to B. duttonii* genome.

### Interspecies analysis

**Figure 1:**
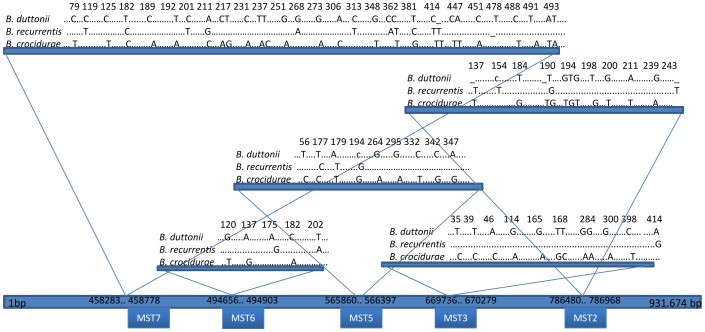
Distribution of spacers among the chromosome of *B. duttonii* and main differences within each spacer.

Pairwise comparison of the five spacers (Table 2 and figure 1) revealed they had species-specific sequence with interspecies sequence differences relying on single nucleotide polymorphism in 36 (90%) cases, deletion in 3 (7.5%) cases and insertion in 1 (2.5%) case. Comparing *B. duttonii* MST7 with *B. crocidurae* and *B. recurrentis* MST7 yielded 93% and 97% similarity, respectively, whilst comparing *B. crocidurae* MST7 with *B. recurrentis* MST7 showed 93% similarity. The other four spacers yielded pairwise sequence similarity of 97–99% (Table 2). Sequences for each allele of each spacer have been deposited in GenBank under accession number (JQ398815: JQ398841) as well as in our local data base (http://www.ifr48.com).

**Table 2:** Pairwise comparison of each spacer of *B. duttonii, B. recurrentis* and *B. crocidurae*.

Species spacers	*B. duttonii*	*B. recurrentis*	*B. crocidurae*	*B. duttonii*	*B. recurrentis*	*B. crocidurae*	*B. duttonii*	*B. recurrentis*	*B. crocidurae*	*B. duttonii*	*B. recurrentis*	*B. crocidurae*	*B. duttonii*	*B. recurrentis*	*B. crocidurae*
	MST2			MST3			MST5			MST6			MST7		
*B. duttonii*	**99–100**	99	99	**100**	99	98	**98–100**	97–100	98–99	**98–100**	99	98	**100**	97	93–94
*B. recurrentis*		**99–100**	99		**100**	98		**100**	99		**100**	99		**100**	93
*B. crocidurae*		99	**99–100**			**99–100**			**99–100**			**98–100**			**99–100**

Bold characters indicate range of similarity within the species.

### Intra-species analysis

**Figure 2:**
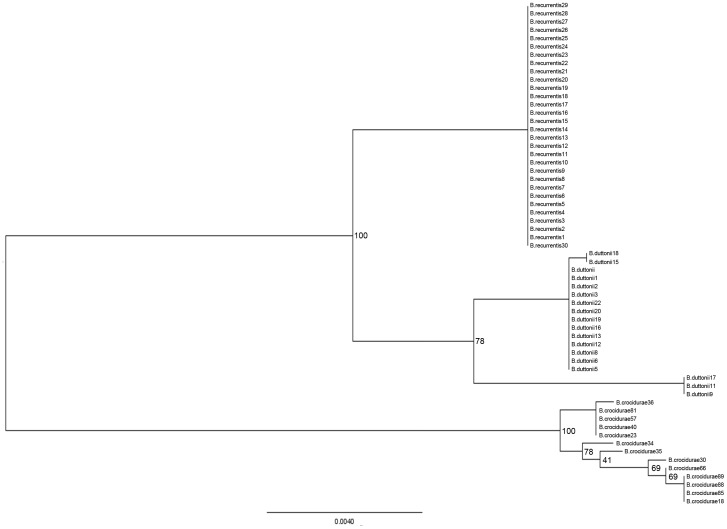
Maximum-likelihood tree based on five intergenic spacers sequences for 61 *Borrelia* strains. To examine the confidence of ML tree, 100 bootstrap replicates were used.

While the concatenation of the five spacers yielded a discrimination index of 0.825,1, this index was of 0.7814 for MST2, 0.6896 for MST6, 0.6749 for MST5, 0.6623 for MST7, and 0.6579 for MST3. Concatenation of the five spacers yielded 8 STs named ST6–ST13 for the 13 *B. crocidurae* samples and the *B. crocidurae* Achema type strain (Table 3; Figure 2). 3 STs named ST1–ST3 for the 18 *B. duttonii* samples and the *B. duttonii* Ly type strain and 2 STs named ST4–ST5 for the 30 *B. recurrentis* samples and the *B. recurrentis* A1 type strain. MST2 sequencing classified latter samples into ST-4 (11 samples) and ST-5 (19 samples) due to the insertion of a G at position 190. The genotype ST-5 represented 47.6% (10 out 21 samples) detected in 1994 and all the nine samples detected in 2011.

**Table 3:** List of spacer types (ST) found in this study.

Species	Strains	ST	MST2	MST3	MST5	MST6	MST7
***B. duttonii***	Bd 9, 11,17	1	1	1	1	1	1
	Bd 1, 2, 3, 5, 6, 8, 12, 13,16, 19,20, 22, Ly	2	2	1	2	2	1
	Bd15, 18	3	2	1	3	2	1
***B. recurrentis***	Br 1,2,3,4,5,6,7,8,9,10,11	4	3	2	4	3	2
	Br12, 13, 14, 15, 16, 17, 18, 19, 20,21,22,23,24,25,26,27,28,29,30,A1	5	4	2	4	3	2
***B. crocidurae***	B.cr18, B.cr89, B.cr88 B.cr85	6	5	3	5	7	4
	B.cr34	7	5	4	5	7	5
	B.cr30	8	6	4	5	4	4
	B.cr35	9	5	4	5	5	3
	B.cr936	10	5	3	5	6	5
	B.cr81 B.cr57 B.cr40, B.cr23	11	5	4	5	6	5
	B.cr66	12	5	4	5	7	4
	Achema	13	7	5	6	8	6

### MST-based phylogenetic analysis of RF *Borrelia*

The phylogenetic tree constructed after concatenation of the five intergenic spacer sequences separated the RF *Borrelia* into three clades, each clade containing only one *Borrelia* species (figure 2). A first clade comprised of all the 30 *B. recurrentis* isolates; a second clade comprised of three groups representing the three *B. duttonii* spacer types and a last clade comprised of 7 *B. crocidurae* spacer types.

## Discussion

PCR-derived data reported herein were interpreted as authentic as the negative controls used in every PCR-based experiment remained negative, all the PCR products were sequenced and experiments yielded reproducible sequences. We therefore established the proof-of-concept that MST could be used for species identification and genotyping of 3 out of 4 cultured RF borreliae (*B. hispanica* was not available for this study) in Africa. MST combines the sensitivity of PCR with unambiguous, portable data yielded by sequencing. Indeed, all the sequences determined are freely available in GenBank and in our local database website at ifr48.com. Therefore, any laboratory with a capacity in PCR-sequencing could easily confirm and compare their data with that reported herein to further increase the knowledge of RF *Borrelia* species and genotypes circulating in African countries.

In the present study, five intergenic spacers were selected from the alignment of *B. crocidurae*, *B. duttonii* and *B. recurrentis* reference genomes, representing approximately ∼0.2% of the total genome length. The spacers were scattered across the chromosome thus representative of the whole genome. Such a multi-target approach offers distinct advantages over the one single locus methods previously used, such as the 16S–23S IGS for typing that may be less representative of the whole genome. Based on this spacer sequencing, a total of 61 RF strains could be separated into 12 STs. Interestingly, we observed that isolates grouped into three clades corresponding to the three *Borrelia* organisms under study. Indeed, MST yielded no overlap between *B. duttonii* and *B. recurrentis* organisms contrary to that observed when using IGS typing [1], [7]. We observed that sequencing MST7 spacer alone accurately discriminated between *B. duttonii* and *B. recurrentis* with 3% sequence divergence, a result not previously achieved. Therefore, sequencing MST7 spacer alone could be used for the molecular identification of RF *Borrelia* in Africa at the species level, but not for genotyping which requires sequencing the four other spacers in addition to MST7.

Further analysis indicated that each one of the three *Borrelia* species under study was comprised of several spacer-types. *B. recurrentis* was the least diverse *Borrelia* comprising of only two very closely related groups. This finding supports the previous genomic analysis that concluded that *B. recurrentis* was a subset of *B. duttonii* [16]. In our study also, there was an inverse correlation between the RF *Borrelia* MST diversity and the reported mortality rate for these RF *Borrelia* [8], [15].

Despite the fact that we tested a small set of *B. crocidurae*, nevertheless we found a high diversity index in this species since 13 *B. crocidurae* samples collected in Senegal yielded 7 MST types and the *B. crocidurae* Achema type strain collected in Mauritania yielded an additional MST type. This first genotyping method for *B. crocidurae* is therefore very promising to probe its geographic repartition as well as potential association of *B. crocidurae* genotypes with vectors. Indeed, four genogroups could be identified in *O. sonrai* ticks collected in Senegal and Mauritania [24]. In this study, *B. crocidurae* flagellin sequence was found identical among the four *O. sonrai* tick groups but the *B. crocidurae* infection rate significantly differed among the four tick groups; MST may help studying such discrepancy and may reveal previously unknown relationships between *B. crocidurae* genotypes and *O. sonrai* genotypes. Moreover, a recent study indicated that *B. crocidurae* may be transmitted by soft tick *Ornithodoros erraticus* in Tunisia, challenging *O. sonrai* as the only *B. crocidurae* vector in West Africa [25]. MST is new laboratory tool to question whether the unexpected higher diversity in *B. crocidurae* than in *B. duttonii* and *B. recurrentis* is linked to a more complex cycle involving several mammals and ticks species.

Present data indicate that MST is offering a new sequencing-based technique for further exploring the identification and genotypes of RF *Borrelia* in vectors and clinical specimens collected in Africa.
