# Correction: Multispacer Sequence Typing Relapsing Fever Borreliae in Africa

**DOI:** 10.1371/annotation/5b575a3d-79c6-4450-9410-225e554da42d

**Published:** 2012-06-22

**Authors:** Elbir Haitham, Gregory Gimenez, Cheikh Sokhna, Kassahun Desalegn Bilcha, Jemal Ali, Stephen C. Barker, Sally J. Cutler, Didier Raoult, Michel Drancourt

The first author's name was spelled incorrectly. The correct name is: Haitham Elbir. The correct citation is: Elbir H, Gimenez G, Sokhna C, Bilcha KD, Ali J, et al. (2012) Multispacer Sequence Typing Relapsing Fever Borreliae in Africa. PLoS Negl Trop Dis 6(6): e1652. doi:10.1371/journal.pntd.0001652

